# Improving Output Performance of a Resonant Piezoelectric Pump by Adding Proof Masses to a U-Shaped Piezoelectric Resonator

**DOI:** 10.3390/mi12050500

**Published:** 2021-04-29

**Authors:** Jian Chen, Wenzhi Gao, Changhai Liu, Liangguo He, Yishan Zeng

**Affiliations:** School of Mechanical Engineering, Hefei University of Technology, Hefei 230009, China; chenjian23@hfut.edu.cn (J.C.); liuchanghai@hfut.edu.cn (C.L.); helg@hfut.edu.cn (L.H.); ysz33@126.com (Y.Z.)

**Keywords:** piezoelectric pump, resonance drive, proof mass, symmetric structure

## Abstract

This study proposes the improvement of the output performance of a resonant piezoelectric pump by adding proof masses to the free ends of the prongs of a U-shaped piezoelectric resonator. Simulation analyses show that the out-of-phase resonant frequency of the developed resonator can be tuned more efficiently within a more compact structure to the optimal operating frequency of the check valves by adjusting the thickness of the proof masses, which ensures that both the resonator and the check valves can operate at the best condition in a piezoelectric pump. A separable prototype piezoelectric pump composed of the proposed resonator and two diaphragm pumps was designed and fabricated with outline dimensions of 30 mm × 37 mm × 54 mm. Experimental results demonstrate remarkable improvements in the output performance and working efficiency of the piezoelectric pump. With the working fluid of liquid water and under a sinusoidal driving voltage of 298.5 V_pp_, the miniature pump can achieve the maximum flow rate of 2258.9 mL/min with the highest volume efficiency of 77.1% and power consumption of 2.12 W under zero backpressure at 311/312 Hz, and the highest backpressure of 157.3 kPa under zero flow rate at 383 Hz.

## 1. Introduction

Piezoelectric pumps have been widely studied in recent years due to their attractive advantages of compact structure, good reliability, low power consumption, high efficiency, precise control ability, fast response, and no electromagnetic influence [1,2]. They have shown extensive application prospects in biomedical treatment, fuel delivery systems, micro-electronic devices, and robots [1,3,4,5].

Piezoelectric pumps basically utilize the reciprocating deformation of piezoelectric actuators to transport liquid [6], and can be mainly divided into reciprocating pumps [7], peristaltic pumps [8], traveling wave pumps [9], centrifugal pumps [10], and some new kinds of pumps [11,12], according to different working principles. Among them, reciprocating pumps are most reported in literatures, including diaphragm pumps and piston type pumps. They are operated based on the volume change of a reservoir with two openings.

In order to improve output performances of the piezoelectric pumps, it is favorable that the piezoelectric vibrator works at the resonant frequency, which will amplify its vibrating amplitude to drive the chamber diaphragm [13,14,15]. Many researchers have tried to optimize the resonance mechanism of piezoelectric pump structure for better performance. Park attached an additional mass to the free end of a piezoelectric stack to enlarge its amplitude of displacement at the resonant point when oscillating the diaphragm of the pump chamber [16]. Ham used a hinge-lever amplification mechanism to amplify the movement of a piezoelectric stack for driving a diaphragm pump at the resonant frequency of 250 Hz [17]. The pump achieved a no-load flow rate of 600 mL/min and maximum output pressure of 6.8 kPa. Simple structure piezoelectric cantilevers have also been utilized to vibrate diaphragm pumps resonantly in different forms [18,19]. Wang proposed a folded piezoelectric vibrator with uniform strain distributions for a high flow rate and high pressure micropump [20]. The folded vibrator worked at the resonant frequency of 361 Hz, and the micropump obtained the maximum flow rate of 118 mL/min and maximum backpressure of 22.8 kPa under a driving voltage of 120 V_pp_.

Since the power density of the piezoelectric element is proportional to its working frequency [21], the operating frequency of the piezoelectric vibrator should be increased. However, the phase lag of check valves has hindered the increase of the operating frequency of the piezoelectric pumps [1,22]. On the one hand, developing check valves with high frequency responses is imperative for high-performance piezoelectric pumps [23,24]; on the other hand, the resonant frequency of the piezoelectric vibrator should be high enough as long as the check valves can operate well [25].

In a previous study, we proposed a U-shaped piezoelectric resonator (UPR) for driving two diaphragm pumps simultaneously under the out-of-phase vibration mode, and a prototype achieved the maximum flow rate of 1660 mL/min and highest backpressure of 85 kPa [26]. In this study, we attempt to further improve the capability of the UPR by adding proof masses to the free ends of its prongs, constructing a folded U-shaped piezoelectric resonator (FUPR). It is supposed that the resonant frequency of the FUPR can be tuned more efficiently to the optimal operating frequency of the check valves by adjusting the sizes of the proof masses in a more compact structure. Since both the FUPR and the check valves can operate at the best condition, and larger torques can be provided by the lumped masses to drive the diaphragm pumps, dramatic performance improvement and smaller volume size can be expected at the same time in a piezoelectric pump by utilizing the FUPR.

## 2. Design and Operation Principle of the Pump

### 2.1. Structure of the Pump

**Figure 1:**
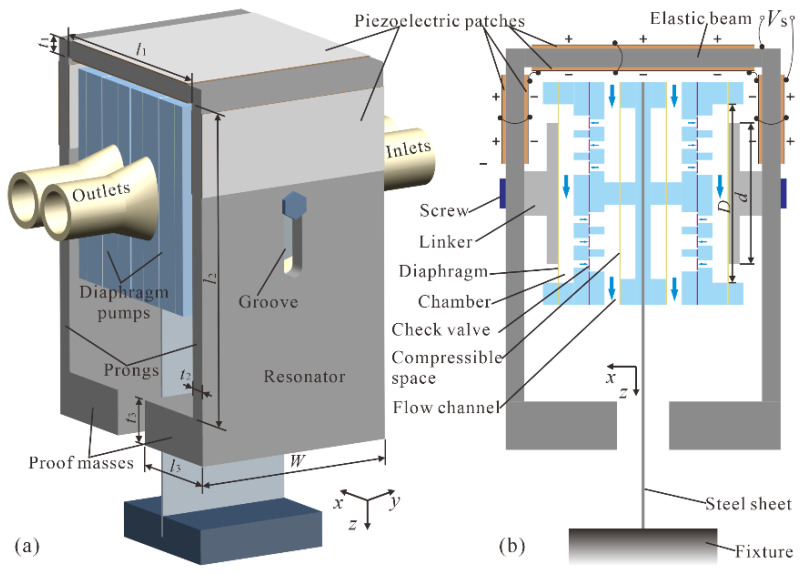
Structure of the proposed piezoelectric pump: (**a**) 3D model; (**b**) Side sectional view.

The structure of the proposed piezoelectric pump is shown in Figure 1. It consisted of the FUPR and two mirror placed diaphragm pumps. The FUPR was constructed by adding proof masses to the free ends of the prongs of a formerly developed UPR. Six piezoelectric patches were adhered to both surfaces of the elastic beam and the prongs of the FUPR, which were polarized and connected to form three parallel piezoelectric bimorphs. The piezoelectric patches on the same surface (inner or outer surface) can be electrically excited to deform synchronously, while the piezoelectric patches on opposite surfaces will deform asynchronously. Thus, a sinusoidal voltage can drive the three piezoelectric bimorphs to bend simultaneously and wobble the two prongs out of phase, which can vibrate the two diaphragm pumps to dispense and absorb liquid periodically and synchronously.

**Figure 2:**
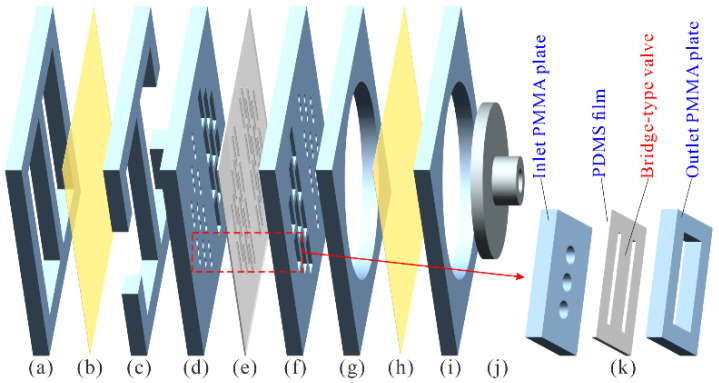
Exploded view of the diaphragm pump: (**a**) Polymethylmethacrylate (PMMA) compressible space seat plate; (**b**) Kapton thin film; (**c**) PMMA flow channel plate; (**d**) PMMA inlet/outlet valve seat plate; (**e**) Polydimethylsiloxane (PDMS) valve thin film; (**f**) PMMA outlet/inlet valve seat plate; (**g**) PMMA chamber seat plate; (**h**) Kapton diaphragm; (**i**) PMMA chamber fix plate; (**j**) Titanium alloy (TC4) linker; (**k**) a check valve unit.

The diaphragm pump adopts a laminated structure, and its exploded view is demonstrated in Figure 2. Four functional units were contained in the diaphragm pump: compressible spaces (Figure 2a,b), inlet/outlet flow channels (Figure 2c), check valve arrays (Figure 2d–f), and a pump chamber (Figure 2g–i). Two compressible spaces are constructed using a piece of Kapton polyimide film (Figure 2b) covering two rectangular holes in a polymethylmethacrylate (PMMA) plate (Figure 2a). The compressible spaces can smoothen the high-frequency pulsation of liquid flow outside the pump chamber, suppress the liquid inertial load, and improve the piezoelectric pump’s output performance significantly [20,26]. The inlet and outlet flow channels were formed by a PMMA flow channel plate (Figure 2c) inserted between the compressible spaces and the check valve arrays. The inlet and outlet check valve arrays were assembled by using two patterned PMMA plates (Figure 2d,f) with rectangular and circular holes to clamp a piece of polydimethylsiloxane (PDMS) thin film with bridge-type valve patterns (Figure 2e). The pump chamber was made of a PMMA chamber seat plate (Figure 2g) sealed by a piece of Kapton diaphragm (Figure 2h) and fixed by a PMMA chamber fix plate (Figure 2i). A titanium alloy (TC4) linker (Figure 2j) was adhered to the chamber diaphragm (Figure 2h), and two identical diaphragm pumps were glued back to back and screwed to the prongs of the FUPR through the TC4 linkers (Figure 1).

A check valve unit from the check valve arrays is illustrated in Figure 2k, which is constructed by a PDMS film sandwiched between two PMMA plates. The inlet PMMA plate was patterned with three circular holes of 1 mm diameter, the outlet PMMA plate was patterned with a rectangle hole of 2 mm × 5 mm in size, and the PDMS film was engraved with two slits to form a bridge-type valve of approximately 1.5 mm × 5 mm size. The bridge-type valve can cover the three circular holes but can deform freely in the rectangular hole. As a result, liquid flow from the circular holes can open the bridge-type valve and flow to the rectangle hole, while liquid flow from the rectangle hole will push the bridge-type valve to cover the circular holes and be blocked from flowing to the circular holes.

### 2.2. Operation Principle

**Figure 3:**
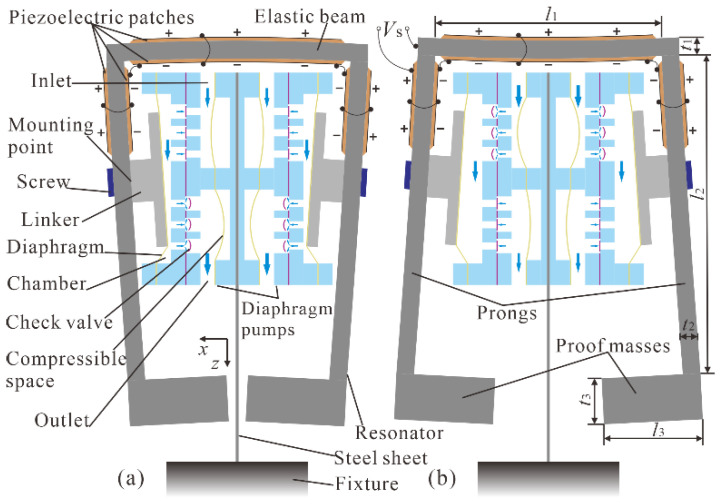
Operation principle of the piezoelectric pump: (**a**) Dispensing mode; (**b**) Absorbing mode.

The operation principle of the piezoelectric pump driven by the FUPR is presented in Figure 3. Under the driving voltage, the two prongs of the FUPR will wobble out of phase. When the prongs vibrate toward each other in the dispensing mode (Figure 3a), the linkers push the chamber diaphragms inwards to the chambers and decrease the volumes of the pump chambers. As a result, the pressures in the pump chambers increase, inlet valves close, outlet valves open, and the liquid inside the chambers is squeezed out into the outlet channels. Alternatively, when the prongs vibrate away from each other in the absorbing mode (Figure 3b), the linkers pull the chamber diaphragms outwards from the chambers and increase the volumes of the pump chambers. Consequently, the pressures in the pump chambers decrease, outlet valves close, inlet valves open, and the liquid inside the inlet channels is sucked into the pump chambers. The dispensing mode and the absorbing mode will proceed alternatively to form a continuous liquid transportation from the inlet to the outlet if the FUPR is excited by a periodic alternating current (AC) voltage.

In the piezoelectric pump, electrical energy was converted into mechanical vibration energy by the FUPR, while mechanical vibration energy was converted into fluid flow energy by the two diaphragm pumps. As the vibrator of the diaphragm pumps, the working condition of the FUPR plays an important role in transporting energy; and the resonance drive of the FUPR under its out-of-phase vibration mode enlarges the vibration amplitude and force that can actuate the diaphragm pumps.

## 3. Simulation Analysis

### 3.1. Modal Analysis

The UPR and FUPR resemble a tuning fork, and the out-of-phase vibration mode was utilized due to its high stability, low energy loss, and good reliability. An approximate analytical solution of the out-of-phase resonant frequency of a tuning fork was $f=(1.76t_{2}/2\pi l_{2}^{2})\sqrt{E/\rho }$, where *E* is the Young modulus of the material, *ρ* is the material density, *l*_2_ is the length of the prongs, and *t*_2_ is their thickness [27]. Although the above equation ignored the effect of the elastic beam, it can be used to estimate the variation trends of the resonant frequency with the dimensions of the UPR and FUPR. Based on the above equation and using software ANSYS 18.0 to perform finite element analysis, the out-of-phase resonant frequencies of the UPR and FUPR were tuned effectively to the experimentally verified optimal operating frequency of the check valves, which was about 310 Hz when transporting liquid water.

**Figure 4:**
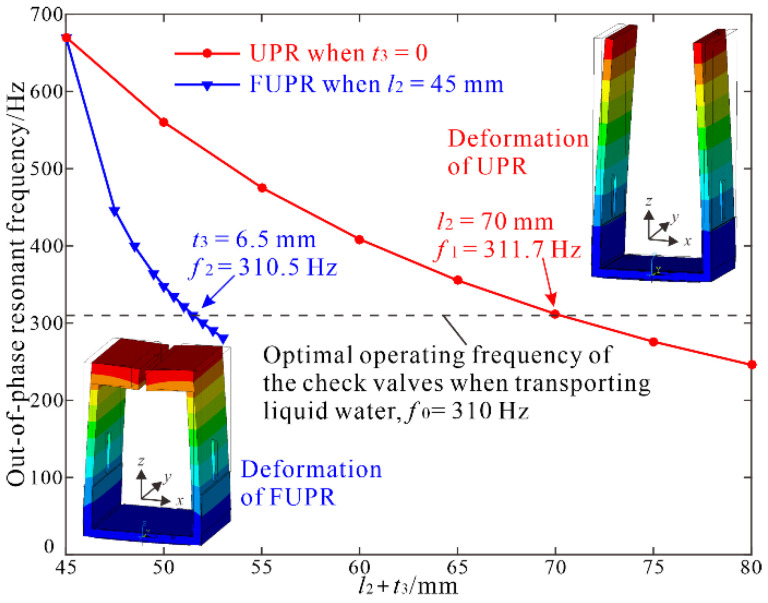
Tuning the out-of-phase resonant frequencies of the U-shaped piezoelectric resonator (UPR) and folded U-shaped piezoelectric resonator (FUPR) by modal analysis.

In order to assemble two diaphragm pumps inside the resonator, set the length of the elastic beam to *l*_1_ = 32 mm, the length of the prongs to *l*_2_ ≥ 45 mm, and the length of the proof masses to *l*_3_ = 15 mm; set the thickness of the elastic beam to *t*_1_ = *t*_2_ = 2.5 mm for good impedance matching between the resonator and the chamber diaphragms; and set the thickness of the piezoelectric patches *t* = 0.2 mm to minimize their influence on the resonant frequency and lower the driving voltage amplitude. Then, modal analyses in ANSYS were conducted for computation of the resonant frequencies of the UPR by changing the value of *l*_2_ when the thickness of proof masses was *t*_3_ = 0; and for computation of the resonant frequencies of the FUPR by changing the value of *t*_3_ when *l*_2_ = 45 mm. The simulation results are shown in Figure 4. It demonstrates that the resonant frequency of the FUPR can be tuned more efficiently to the desired frequency in a smaller volume size. The out-of-phase resonant frequency of the FUPR was tuned to 310.5 Hz when *t*_3_ = 6.5 mm, while the out-of-phase resonant frequency of the UPR was tuned to 311.7 Hz when *l*_2_ = 70 mm.

**Figure 5:**
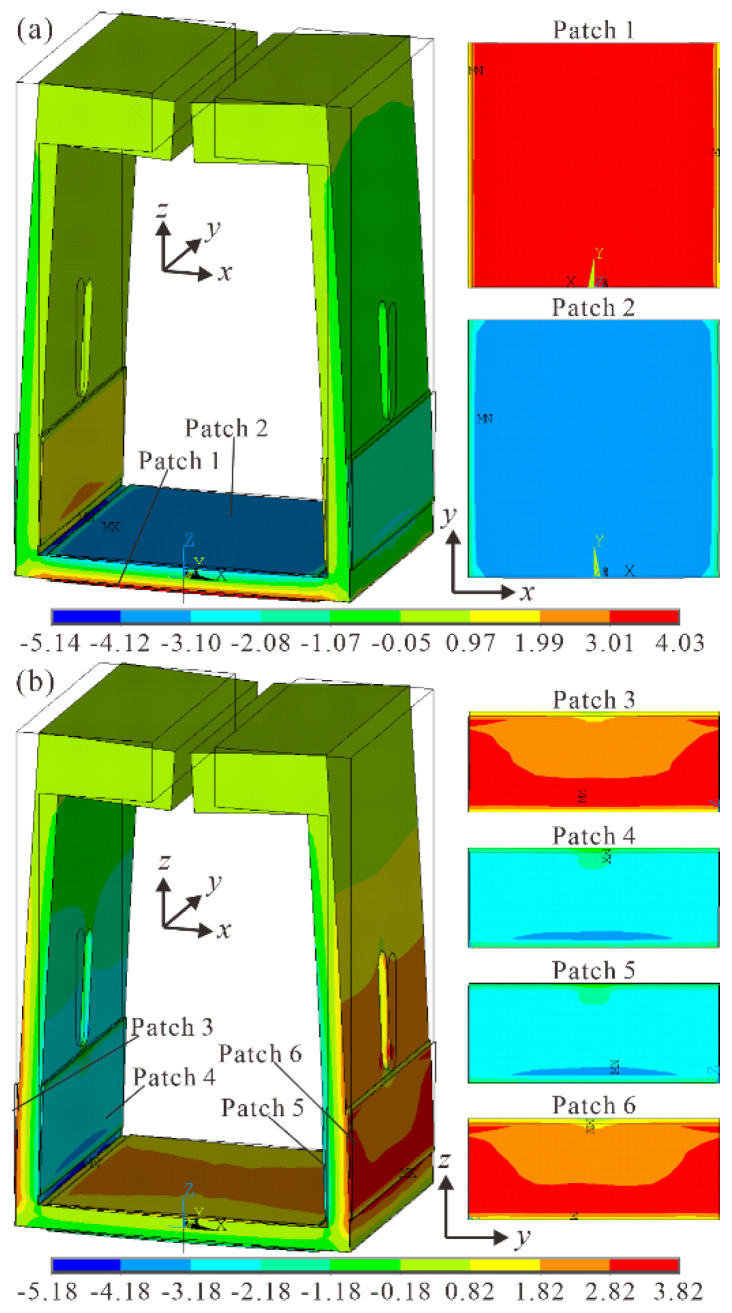
Mechanical strain of the FUPR under out-of-phase vibration mode: (**a**) *x* component of total mechanical strain; (**b**) *z* component of total mechanical strain.

Since the FUPR was bent by the piezoelectric patches attached to it through inverse piezoelectric effect, bending strain distributions of the piezoelectric patches had significant effects on the working efficiency and reliability of the FUPR. In order to explore the bending strain distributions of the piezoelectric patches, *x* component (Figure 5a) and *z* component (Figure 5b) of total mechanical strain were obtained in the modal simulation results. It was seen that maximal and uniform bending strain distributions (along *x* axis) were achieved on the surfaces of piezoelectric patches 1 and 2, while slightly lower bending strain values (along *z* axis) were obtained on the surfaces of piezoelectric patches 3, 4, 5, and 6. The simulation results demonstrated that the piezoelectric patches can fully expand in their bending directions under the same exciting voltage, which can improve the working efficiency and reliability of the resonator remarkably.

### 3.2. Harmonic Analysis

**Figure 6:**
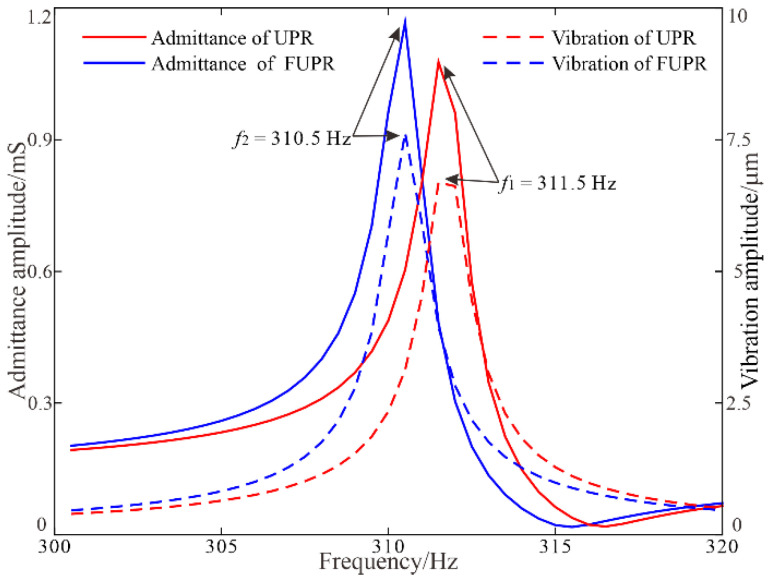
Frequency responses of the UPR and FUPR by harmonic analysis.

To evaluate the frequency characters of the piezoelectric resonators, harmonic analyses were executed in ANSYS under free vibration condition with a driving voltage of 1 V_pp_ and a frequency range from 300 Hz to 320 Hz in steps of 0.5 Hz. Frequency responses of the admittance amplitudes and vibration amplitudes along *x* direction at the mounting point were obtained and displayed (Figure 6). For the FUPR, the resonance curves reached their peaks at 310.5 Hz, while for the UPR, the resonance point was 311.5 Hz, and the peak values of the two resonators reached almost the same level considering the finite frequency step in the analyses.

## 4. Fabrication and Experiments

### 4.1. Fabrication of the Pump

The FUPR and diaphragm pumps used to construct the piezoelectric pump were fabricated separately and assembled subsequently. Firstly, the framework of the resonator was made of SUS 304 stainless steel using high-precision wire-cut electrical discharge machining. Two grooves were cut in the middle line of the prongs for the mounting of the diaphragm pumps. Then, six piezoelectric patches (PZT-4, *d*_33_ = 340 pC/N, *d*_31_ = −100 pC/N) purchased from Hunan Jiayeda Electronics Co., Ltd. (Hunan, China) were adhered to the surfaces of the resonator using epoxy adhesive (DP460), as demonstrated in Figure 1.

A diaphragm pump was fabricated from six PMMA plates (Figure 2a,c,d,f,g,i), two pieces of Kapton thin film (Figure 2b,h), and a piece of PDMS thin film (Figure 2e). The PMMA plates were patterned with designed circular or rectangular holes, according to different functionalities using computer numerical control (CNC) laser processing. A piece of Kapton thin film (Figure 2b) was adhered to a PMMA compressible space seat plate (Figure 2a), constructing the unit of compressible spaces; then, it was adhered to a PMMA flow channel plate (Figure 2c), building one side wall of the flow channels. A piece of PDMS thin film (Figure 2e) was firstly sandwiched between two PMMA valve seat plates (Figure 2d,f), then carved with narrow slits forming the bridge-type inlet/outlet valve arrays. A PMMA pump chamber seat plate (Figure 2g) was covered by a piece of Kapton diaphragm (Figure 2h) and fixed by a piece of PMMA chamber fix plate (Figure 2i), forming the unit of the pump chamber. Then, the above pump units were adhered to each other in the sequence shown in Figure 2, constructing a diaphragm pump. A linker (Figure 2j), made of titanium alloy TC4 with CNC lathe, was attached to the chamber diaphragm (Figure 2h) for assembly with the FUPR. During the fabrication process, epoxy adhesive (DP460) was used for gluing, sealing, and strengthening the laminated layers of the diaphragm pump.

**Figure 7:**
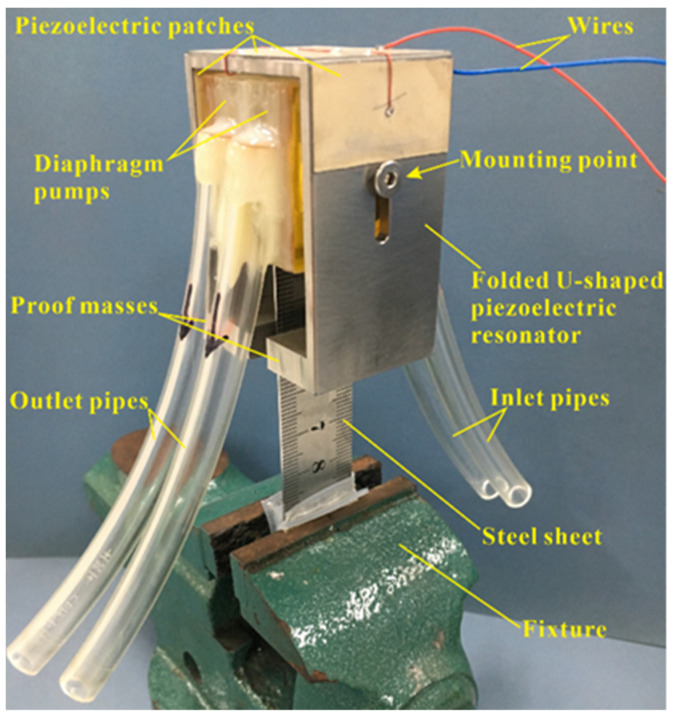
Photograph of a prototype piezoelectric pump.

Two diaphragm pumps were glued back-to-back and screwed to the prongs of the FUPR. A prototype piezoelectric pump was clamped to the fixture via a flexible steel sheet connecting the diaphragm pumps, as exhibited in Figure 7. The dimensions of the piezoelectric pump are summarized in Table 1, while the main properties of the materials used are listed in Table 2.

### 4.2. Experimental Setup

**Figure 8:**
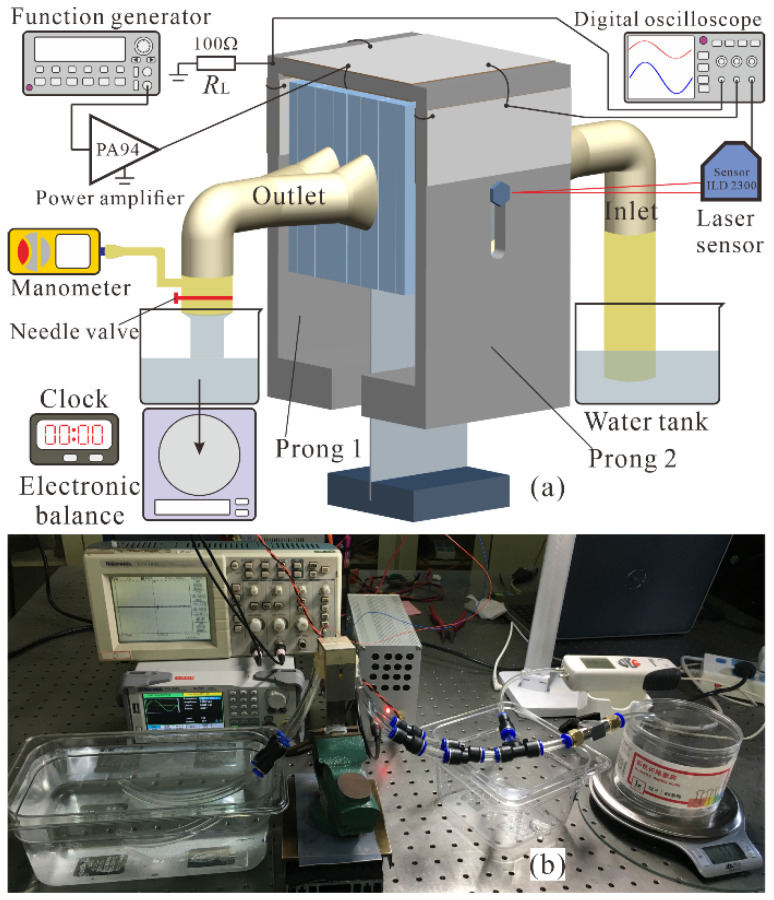
(**a**) Schematic diagram and (**b**) photograph of the experimental setup.

The performance of the prototype piezoelectric pump was investigated experimentally with the setup shown in Figure 8. Tap water was used as the working liquid and the experimental tests were carried out at an ambient temperature of about 20 °C. A sinusoidal voltage was generated by a function generator (DG1022, RIGOL, Beijing, China), and then magnified by a homemade power amplifier (PA94, Apex Microtechnology, Tucson, AZ, USA) to drive the piezoelectric patches. A digital oscilloscope (TDS2012, Tektronix, Beaverton, OR, USA) monitored the driving voltage. A commercial laser displacement sensor (optoNCDT ILD 2300-2, Micro-Epsilon, Ortenburg, Germany) was utilized to detect the vibrating amplitudes of the chamber diaphragms at the mounting points of the linkers on the prongs. An electronic balance was used to measure the output liquid mass during a time interval, and the flow rate was calculated, given the density of tap water (1000 kg/m^3^). Backpressure of the pump was monitored by a digital manometer. To obtain the power consumption of the piezoelectric pump, a sampling resistor, *R*_L_ (100 Ω), was inserted between the piezoelectric patches and the ground, and the voltage across the sampling resistor was monitored and divided by 100 Ω to get the current in the circuit, which was further integrated by the voltage across the piezoelectric patches over one cycle to calculate the power consumption in the piezoelectric elements. In order to decrease influence of experimental error and present the repeatability of the prototype pump, the vibration amplitude, flow rate, and backpressure were measured three times, the average values were taken as the final results, and the standard deviations were calculated as error bars.

## 5. Results and Discussions

### 5.1. Experimental Results

**Figure 9:**
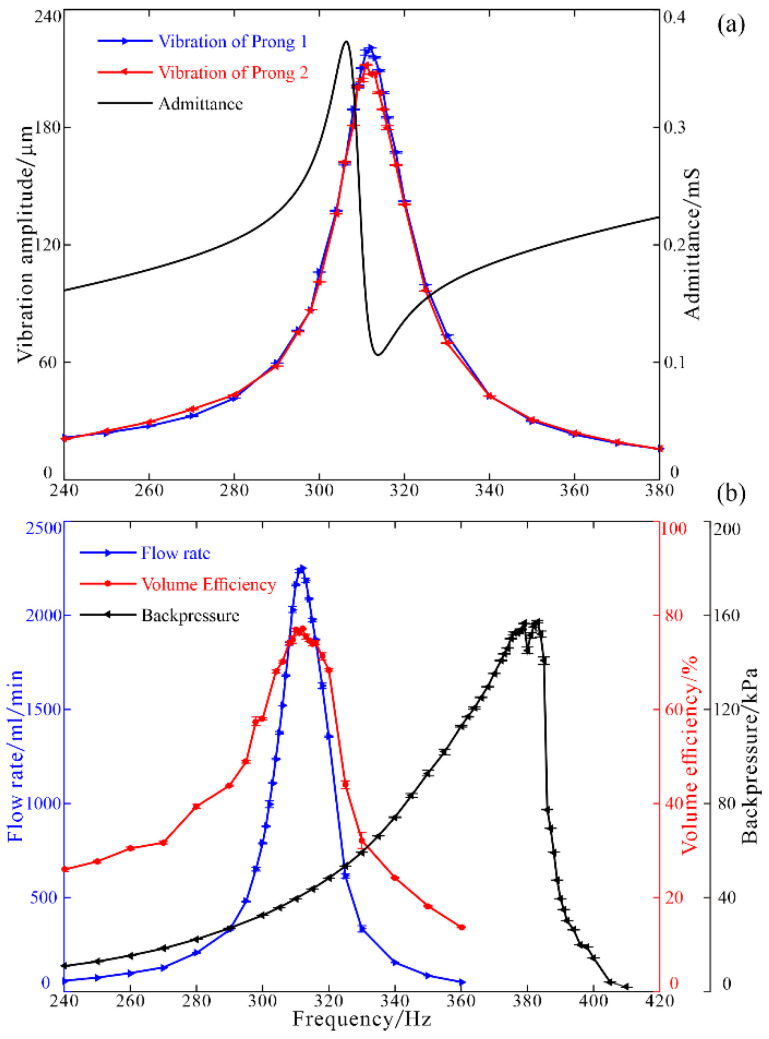
Frequency characters of (**a**) admittance of the pump and vibrations of the prongs; (**b**) flow rate, volume efficiency, and backpressure.

Frequency characters of the prototype piezoelectric pump were measured and shown in Figure 9. First of all, the admittance, without liquid in the pump, was measured by an impedance analyzer (IM3570, HIOKI, Nagano, Japan), and the resonant frequency was 306.2 Hz (Figure 9a). Then, frequency responses of the vibration amplitudes (peak-peak value) of the prongs, flow rate, and backpressure were investigated to get overall performance characteristics of the piezoelectric pump. The driving voltage was set at a constant value of 298.5 V_pp_, the vibration amplitudes and flow rate were measured under zero backpressure, and the backpressure was measured under zero flow rate.

In order to verify that the two pumps were symmetrically actuated by the FUPR and to minimize the calculation error of volume efficiency, the vibration amplitudes of the two prongs (*A*_1_ and *A*_2_) were detected, respectively. The results demonstrated that the vibration amplitudes of the two prongs showed good consistency, with the peak amplitude of prong 1 reaching 220.5 μm at 312 Hz and the peak amplitude of prong 2 reaching 211.9 μm at 311 Hz (Figure 9a). Predictably, the no load flow rate follows the same trend as the vibrating amplitudes (Figure 9b). It showed that the piezoelectric pump can deliver water at a frequency range from 240 to 360 Hz, and the maximum flow rate reached 2252.3 mL/min at 312 Hz. The volume change, Δ*V*, of the two pump chambers in one cycle can be estimated by using the volume formula of frustum of a cone:

(1) $$\Delta V=\frac{\pi }{12}(A_{1}+A_{2})(D^{2}+d^{2}+Dd),$$

where *D* and *d* are diameters of the pump chamber and the linker disk, respectively (Table 1). The theoretical flow rate, *Q*_t_, is calculated by multiplying the volume change Δ*V* with corresponding oscillating frequency *f*:

(2) $$Q_{t}=\Delta V\cdot f.$$

Then, the volume efficiency *η*_v_ can be obtained by dividing the actual flow rate, *Q*, by the theoretical flow rate, *Q*_t_:

(3) $$\eta_{v}=\frac{Q}{Q_{t}}.$$

The peak volume efficiency was 77.1% at 312 Hz (Figure 9b), which validates the superiority of the resonance drive. The backpressure under zero flow rate was monitored and shown in Figure 9b. It can be found that the backpressure reaches its peak value of 157.3 kPa at a higher resonant frequency of 383 Hz.

It can be predicted that increasing the driving voltage amplitude will improve the output performances of the piezoelectric pump. However, the driving voltage amplitude was limited by the poling field, coercive electric field, and thickness of the piezoelectric patches. In this study, the poling field of PZT-4 was 2 kV/mm, the coercive electric field was about 1/3 of the poling field, and the thickness of the piezoelectric patches was *t* = 0.2 mm, thus, the piezoelectric patches can be operated at a voltage range of −133 V to 400 V. In the experiments, the maximum driving voltage amplitude was set to be approximately 300 V_pp_ with no DC bias.

**Figure 10:**
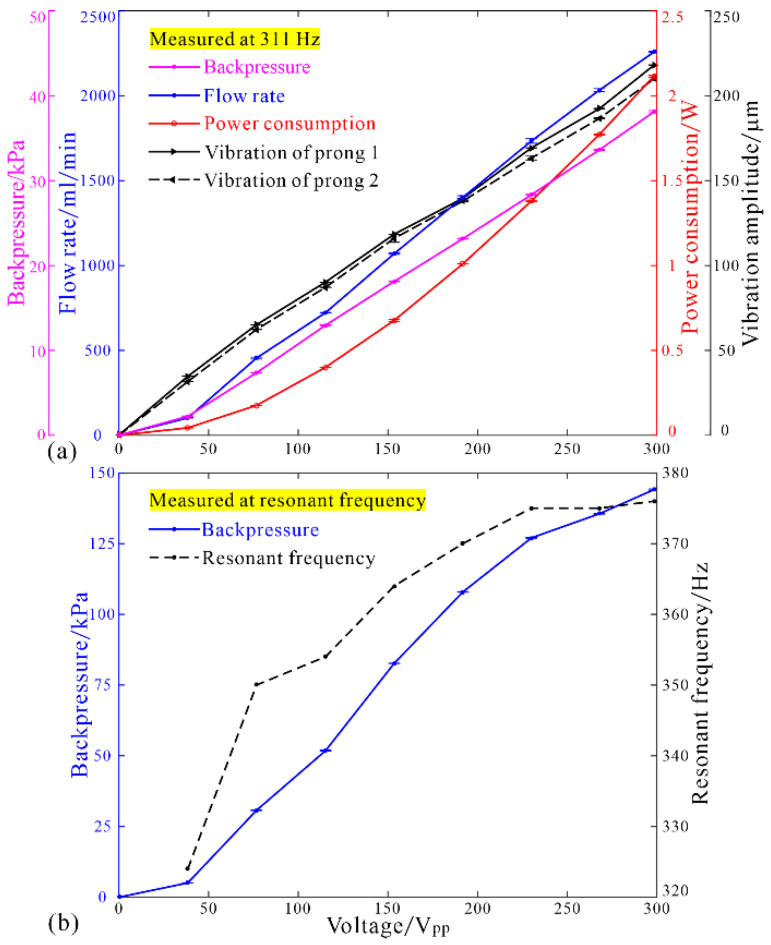
Influences of the driving voltage on (**a**) flow rate, vibration amplitudes, power consumption, and backpressure at 311 Hz; (**b**) backpressure at resonant frequency.

Firstly, the driving frequency was set at a constant value of 311 Hz, the vibration amplitudes, flow rate, and power consumption were measured under zero backpressure, and the backpressure was measured under zero flow rate. The results in Figure 10a showed that the vibration amplitudes of the prongs, flow rate, power consumption, and backpressure increased approximately linearly with the driving voltage amplitude. Under the driving voltage of 298.5 V_pp_ at 311 Hz, the vibration amplitude of prong 1 reached 218.0 μm, the vibration amplitude of prong 2 reached 210.0 μm, the flow rate reached 2258.9 mL/min, the power consumption reached 2.12 W, and the backpressure reached 38.1 kPa. Secondly, the highest backpressure under zero flow rate was recorded by adjusting the driving frequency to the corresponding resonant frequency at a certain exciting voltage amplitude. In this case, the backpressure increased linearly, but faster with the driving voltage, and the resonant frequency increased irregularly with the driving voltage until tending to a constant value (Figure 10b). In the end, the backpressure reached 144.1 kPa at 376 Hz under the driving voltage of 298.5 V_pp_.

**Figure 11:**
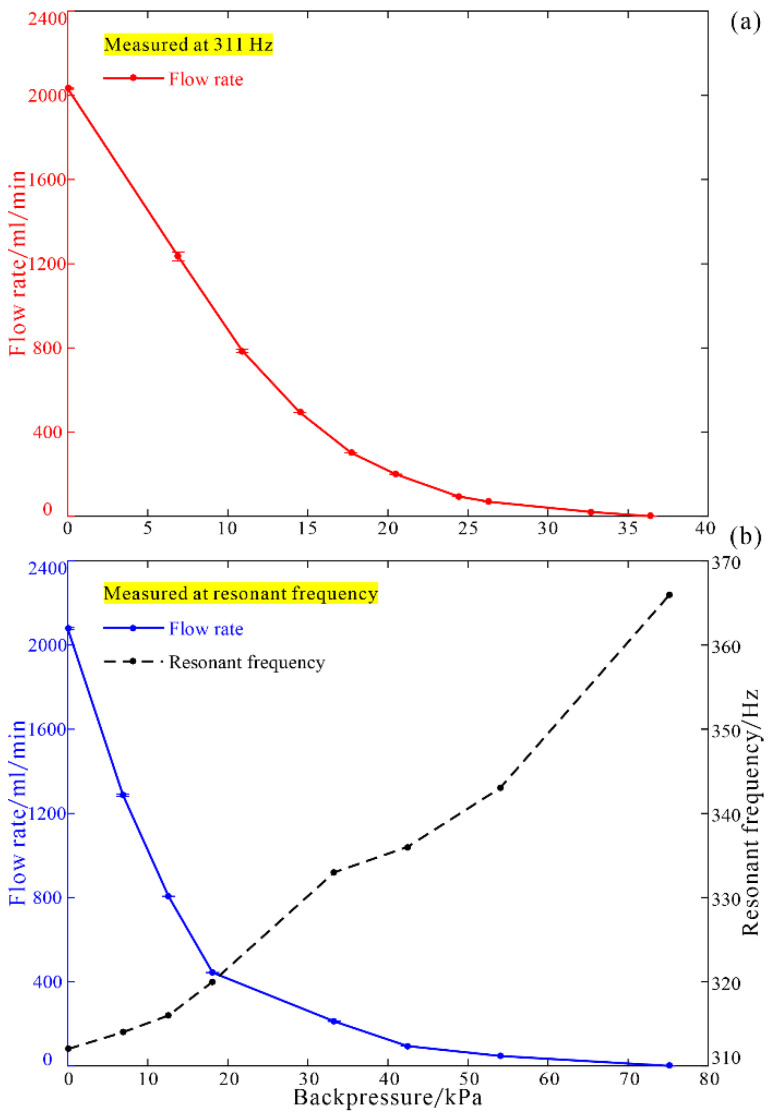
Load characters of flow rate (**a**) at 311 Hz, and (**b**) at resonant frequency.

Load characters of the piezoelectric pump were tested using a needle valve adjusting the backpressure at the outlet pipes under a constant driving voltage of 298.5 V_pp_. The flow rate will decrease with the increase of the backpressure (Figure 11). When the driving frequency was set at a constant value of 311 Hz, the maximum flow rate was 2032.0 mL/min, and the maximum backpressure was 36.4 kPa (Figure 11a). When the driving frequency was manually adjusted to the resonant points under different backpressures, the maximum flow rate was 2073.4 mL/min at 312 Hz and the maximum backpressure reached 75.2 kPa at 366 Hz (Figure 11b). Evidently, the resonant frequency of the piezoelectric pump increased with the increase of backpressure. A smaller value of highest backpressure and a lower corresponding resonant frequency detected here may be owing to the longer elastic pipes attached to the outlets of the pumps.

### 5.2. Discussions

The experimental results demonstrate that the developed piezoelectric pump can achieve both large flow rate and high backpressure while keeping a compact structure. When compared with the previous piezoelectric pump driven by the UPR [26], the piezoelectric pump driven by the FUPR achieved a volume size reduction of 25.5%, a flow rate improvement of 34.9%, a backpressure improvement of 84.4%, and a volume efficiency improvement of 94.7% under almost the same operating frequency and exciting voltage.

The proposed piezoelectric pump has many outstanding features, such as simple structure, separable design, ease of fabrication, free of sliding components, and uniform strain distribution, which ensures that the piezoelectric pump has high power density, high efficiency, good reliability, and a long working life. Although the overall size of the developed piezoelectric pump is still relatively large, it can be easily miniaturized or even microminiaturized, yet retain competitive output capabilities to meet various demands in application areas of medical treatments, chemical and biological analysis systems, fuel delivery for fuel cells, and microelectronics cooling systems. In addition, the primary resonant frequency of a piezoelectric pump can be tuned conveniently to meet the best working frequency range of different types of check valves; and closed-loop control circuits can be developed to enable automatic tracking of the resonant frequency and enhance working stability and precise control of output liquid of the piezoelectric pump.

## 6. Conclusions

In order to improve the output performance of a resonant piezoelectric pump, proof masses were added to the free ends of the prongs of a U-shaped piezoelectric resonator, constructing the proposed folded U-shaped piezoelectric resonator. Simulation analyses verified that the out-of-phase resonant frequency of the FUPR can be tuned more efficiently to the optimal operating frequency of the check valves by adjusting the thickness of the proof masses in a smaller volume size. The FUPR was utilized with two diaphragm pumps to construct a separable piezoelectric pump, and the output performances of the pump were experimentally tested. The results demonstrated that, under a sinusoidal driving voltage of 298.5 V_pp_, the prototype piezoelectric pump can transport liquid water to the maximum flow rate of 2258.9 mL/min with the highest volume efficiency of 77.1% and power consumption of 2.12 W under zero backpressure at 311/312 Hz, or generate the highest backpressure of 157.3 kPa under zero flow rate at 383 Hz. In summary, this study proposed a FUPR for driving diaphragm pumps. The resonant frequency of the FUPR can be tuned conveniently to adapt to the optimal operating frequencies of various types of check valves while keeping a compact size, and remarkable output performance improvement can be achieved in the piezoelectric pump.

## Figures and Tables

**Table 1:** Dimensions of the piezoelectric pump.

Parameter	Symbol	Value (mm)
Piezoelectric pump size	*W* × *L* × *H*	30 × 37 × 54
Elastic beam length	*l*_1_	32
Elastic beam thickness	*t*_1_	2.5
Prong length	*l*_2_	45
Prong thickness	*t*_2_	2.5
Proof mass length	*l*_3_	15
Proof mass thickness	*t*_3_	6.5
PMMA plate size	*W*_1_ × *L*_1_ × *T*_1_	30 × 30 × 2
Chamber diameter	*D*	24
Linker disk diameter	*d*	19
Kapton film size	*W*_2_ × *L*_2_ × *T*_2_	30 × 30 × 0.05
PDMS film size	*W*_3_ × *L*_3_ × *T*_3_	30 × 30 × 0.2

**Table 2:** Material properties of the piezoelectric pump.

Material	Density (kg/m^3^)	Young’s Modulus (GPa)	Poisson’s Ratio
PZT-4	7450	79	0.31
SUS 304 stainless steel	7820	200	0.29
TC4	4430	110	0.34
PMMA	1190	3.16	0.32
Kapton	1420	2.55	0.34
PDMS	956	7.5 × 10^−4^	0.45

## Data Availability

The data presented in this study are available upon reasonable request from the authors.
